# Topics of the Month

**Published:** 1895-10

**Authors:** 


### TOPICS OF THE MONTH.

WITHIN the mystic realm of that eminently scientific no-code organization, known as the Mississippi Valley Medical Association, there is a social club which is recognised everywhere by the euphemystic title of Chutmucks. Membership in this latter club can only be obtained through the gateway of the former mentioned body. The meetings of both are held once a year, and the Chutmucks are in perpetual session from the opening to the close of the medical society of which they form so important a counterpart.

The proceedings at the late meeting in Detroit, in so far as the Chutmucks were concerned, were varied from the common in a most pleasant manner, which we may best describe by quoting the report as published in the *New York Medical Journal* :

THE CHIEF OF THE "CHUTMUCKS."—Dr. Isaac N. Love, of St. Louis, is recognised as the chief of a semi-organised social coterie known as the "Chutmucks." Among his peculiarities has heretofore been that of not carrying a watch ; to use his own words, he would not be "a slave to time." Time has been called on him, however, and he now carries a watch that was given to him in Detroit last week, during the meeting of the Mississippi Valley Medical Association. Among other inscriptions, the watch bears this : "To the Chief of the Chutmucks, from his Loyal Braves." On the occasion of the presentation, speeches were made by Dr. Charles A. L. Reed, of Cincinnati, and Mr. William J. Evans, of New York. Mr. Evans is reported in the *Detroit Free Press* as having said :

"Those who do not know, and do not know that they do not know, these are fools ; leave them. Those who do not know and know that they do not know, these are children ; teach them. Those who know and do not know that they know, these are asleep ; arouse them. Those who know and know that they know, these are wise ; follow them. Tonight, gentlemen, we are assembled to honor one who knows, one who makes men friends and women sweethearts ; one who is the daily mirror of a broad fraternal love, a man who, having crossed the ocean with unequaled gifts as a gentleman, a scholar and a Chutmuck, saw, heard and felt those things which go to make the plenitude of men on foreign shores, and, after writing on their maps the word 'inadequate,' has returned to those who love the common ground on which he helps them stand ; and with him here

" We now can think, and think aright ;
We now can see with mental sight
That love strives ever to become
Of plenitude in man the sum."

Hail to the CHIEF OF THE CHUTMUCKS ! Long may he wave !

---

THE *Buffalo Express*, of Saturday, September 21st, publishes an account of a collision between the Emergency ambulance and a heavily laden dray wagon, at North Division and Oak streets, in this city. While we do not assume to decide as to who was technically at fault in this deplorable accident, in which both the surgeon and driver of the ambulance were seriously injured, we yet insist that it is strongly in evidence that the ambulance was being driven at an unnecessary rate of speed. Five minutes' difference in the arrival of an ambulance at a point where its services are required does not materially influence the injured, while the difference in speed necessary to cover this five minutes does greatly jeopardise life and limb.

This accident teaches an important lesson and furnishes another argument for the establishment of the trolley ambulance, the advantages of which were fully set forth in an illustrated editorial, published in the issue of the JOURNAL for August, 1895.

Let the day be hastened when the electric railway ambulance shall take the place of the present dangerous commercial system, until which time let the speed of the horse ambulance be regulated by law.

---

DR. FORBES WINSLOW, a recognised authority on mental diseases, arrived in this country, August 31, 1895, for the purpose of attending the medico-legal congress. Dr. Winslow is the founder and head of the British hospital for mental diseases and is editor of the *Psychological Journal.* He has devoted much time to the investigation of criminal responsibility. His father was pioneer in bringing about a recognition of the insanity plea as a defense in criminal cases. Previous to 1844 it had never been considered, hence, no matter what the mental condition of a criminal happened to be, if convicted, he was punished ; if found guilty of murder, he was executed. In the Macnaughton case, which is well known in English jurisprudence, Dr. Winslow, Sr., was called by the judge to give evidence on the point of the prisoner's alleged insanity. He showed conclusively that Macnaughton was not in his right mind when the crime was committed, and ever since that time the plea of insanity has been recognised in the highest courts.

The visit of Dr. Winslow possesses additional interest from the fact that he has been indefatigable in his efforts to secure the release of Mrs. Maybrick, who has been rendered notorious by her conviction of poisoning her husband and the subsequent agitation started by Dr. Winslow that brought about a commutation of her sentence to life imprisonment. The following, from Dr. Winslow's own lips in a recent conversation, will state his position in the case:

I have investigated the whole matter thoroughly and everything I have discovered confirms my judgment that Mrs. Maybrick was not responsible for her husband's death. I proved conclusively, for one thing, that the small amount of arsenic found in the dead man's body was accounted for by the arsenical medicine administered by Mr. Maybrick's doctor a few days before death took place. I am personally acquainted with that doctor and the information I obtained from him leaves it a certainty that the amount of arsenic prescribed was sufficient to account for the arsenic found in the body afterward. In fact, everything I have ascertained from personal investigation tends to demonstrate the innocence of Mrs. Maybrick.

"Do you think that the government will eventually be converted to the same belief and give her her freedom?" the doctor was asked. "There is no doubt of it," he replied. "The facts in her favor are being so strengthened that I expect she will be out of jail in six months."

---

THE "scientific food" theory has had a setback. It has been weighed in the balances of experience and found wanting. A detachment of the United States army was detailed to try it. Fifty men were sent out for a three days' march with "scientific rations in their haversacks. They had little tablets of highly concentrated essences of coffee and bread, which, it was imagined, would be as serviceable as the bulkier drink and food in their usual form. But, at the end of the first day, three-quarters of them were doubled up with cramps. Their digestive organs could not assimilate the concentrated food, or were not satisfied with it, and the experiment was a failure. It is stated that the men were ordered to keep on to the end of the three days' trial, which seems a needless hardship. Their stomachs and intestines are not likely to take to a diet of chemical tablets any more kindly on the second or third day than on the first.

There is something attractive in the notion of scientific food. In many respects it would be a great gain if men could live on a few pills, or powders, or tablets, instead of bulky masses of bread and meat. It would greatly simplify our domestic economy and give each household its longed-for independence from the tyranny of the cook. But it is impossible. A million or so years hence it may be effected, when man has become physically as different from what he is today as he is today from the mollusk, but not before.

The man of today not merely requires a certain amount of absolute nutriment, but he requires it to be commingled with a certain bulk of other matter which is not nutriment at all. He has not only organs of assimilation, but equally elaborate and important organs of excretion, which demand to be supplied with material for their work.

The argument against scientific food—or, more properly, chemically concentrated food—is, therefore, resolved into a parallel to Pope's lines :

Why has not man a microscopic eye ?
For this plain reason, man is not a fly.

Man cannot exist upon a chemical diet, for the plain reason that he is a man. He has physical requirements and functions which demand food in other form than chemical extracts. Much may, of course, be done by science to improve our food supply, to render it more wholesome, more efficient, perhaps more convenient and portable. Beyond such limits it can scarcely go. Bread is the "staff of life," and, with meat, and fruit, and vegetables and other adjuncts, is apparently destined to remain so to the end of time.—*New York Tribune.*

---

AT LIBERTY, N. Y., (*Maryland Med. Jour.*), there will soon be built a new rural retreat for consumptives, for which purpose $20,000 has been contributed by Mr. J. Pierpont Morgan. The location has a high reputation for salubrity and attractiveness. Its easy accessibility to a large population, needing a sanitary retreat less remote from the metropolitan district than are the Adirondacks, will tend to build it up even more rapidly than that on the Saranac.

---

AT THE Medico-Legal Congress, held in New York, September 4, 5 and 6, 1895, Dr. William Lee Howard, of Baltimore, read a paper entitled Sexual Perversion and Crime, and while there incidentally gave some reasons and facts that called for the regulation of hypnotism by legal action. In the *New York Tribune*, September 15, 1895, Mr. Joseph H. Fussell challenges Dr. Howard's methods of psychological investigations. It appears that Dr. Howard, by means of a hypnotic suggestion, caused an honest man to commit theft and that afterward, when the stolen property was found upon the man, he fell into a cataleptic condition. Dr.

Howard adds that the man's brain was, he believed, permanently injured.

Dr. Howard replies to Mr. Fussell in the *Tribune*, September 20th, in course of which he writes as follows :

I have strongly advocated the legal suppression of the traveling and ignorant mesmeriser. The trail of hysteria that is left among the young women in the towns visited by these persons can only be understood by one who has followed the course of these crude exhibitors. All public demonstrations of hypnotism should be suppressed by legal action, and the courts should take cognisance of citizens being subjugated by any unqualified experimenter, as it does of the unlawful practice of drug prescribing or surgical operations by unqualified and unlegalised persons. It was to enable me to take a decided stand in this matter that I have experimented so far with the various phases of hypnotism. I am satisfied with my facts. It has been done with that scientific zeal and desire for truth that accompany all investigations. If Mr. Fussell thinks that I should have ceased at the point of finding out whether crime could be committed by hypnotic suggestion or not, I beg to differ from him. By doing as I have I hoped to prevent further crime from being committed through this agency, as well as to put a stop to those numerous and silly pleas that are now brought forward in our courts of hypnotic influence. I believe that only a small percentage of subjects can be made to commit crime, but we must take cognisance of that small number.

For a full explanation of this subject I refer Mr. Fussell to my article in the *New York Medical Journal*, March 9, 1895. Others in numerous daily journals throughout the country, as well as Mr. Fussell in the *Tribune*, ask about the legal responsibility of the hypnotiser and the hypnotised. With your kind permission I will answer all through the *Tribune*.

In discussing the question from the point of view of criminal law, we are confronted by two great questions : first, the responsibility of the hypnotiser and, second, the responsibility of the hypnotised. To the first question the answer is simple. The hypnotiser occupies a position akin to that of an accessory before the fact who, under the common law of England, is equally guilty with, and is punished as, a principal ; but in the case of a crime committed by one under hypnotic suggestion, the guilt of the hypnotiser is increased. In the case of principal and accessory there are two wills acting in unison, but in the other the will of the hypnotiser stands alone in the guilt, and if the crime was murder his position is precisely that of one who lets loose a wild animal upon his victim, knowing that the nature of the animal is such that he will surely kill.

From what I have said on the first question it follows as a corollary to the proposition that the second question is answered, by saying that the responsibility of the hypnotised is no greater than that of one who is *non compos mentis ;* but this broad generalization requires to be somewhat qualified, and in this connection I desire to note my dissent from Albert Moll, in his sweeping condemnation of the view of Desjardins, "that a person who commits a crime by post-hypnotic suggestion is punishable, because he might have foreseen the possibility of such suggestion."

This language is, from a legal point of view, objectionable, in that it is vague and liable to misconstruction ; but with certain qualifications it can, I think, be fairly indorsed. If the party hypnotised knew previously that the hypnotiser had this power and was a man of criminal habits and inclinations, and that he himself was subject to hypnotic influence, and yet, while in full possession of his will, he placed himself in such a position as to be within the scope of hypnotic influence, I am strongly of the opinion that a certain degree of legal responsibility attaches to him for any crime he may commit, either under the influence of hypnotic or post-hypnotic suggestion, though to what extent he should be punished I am not prepared to say ; but his position might fairly be held to be somewhat analogous to that of an engineer by whose carelessness a passenger was killed—the absence of criminal intent being the same in either case. If, of course, the one hypnotised has caused the criminal act to be suggested to him, the guilt of both parties would be equal.

Now, having fixed the status of the hypnotiser and the hypnotised before the criminal law as to their respective responsibilities, we come to what I regard as the most difficult problem to solve : how is it to be demonstrated that a given crime was committed by a prisoner while in a state of hypnosis ? Such claims are now frequently being brought before our courts. Such a defense may rightly be interposed, and while evidence might properly be introduced in a homicidal case to show that the prisoner had no motive to kill the deceased, but that A had such a motive, and that A possessed the hypnotic power, and that the prisoner was subject to hypnotic influence, and while such evidence might raise in the minds of the jury such reasonable doubt that they would be forced to acquit the prisoner ; yet, when the picture is reversed and we see A placed at the bar, the prosecution is beset with such difficulties under the rules of evidence, that I do not consider it would be competent for it to prove the responsibility of A by showing that he had exercised the hypnotic power over B at other times, any more than it would be competent for it to prove the guilt of a prisoner by showing that he had been guilty of similar offenses at other times. This evidence is always inadmissible except in rebuttal, where the defense has offered in chief evidence, tending to show the previous good character of the accused. While such evidence would undoubtedly tend to carry moral conviction, it would, nevertheless, be legally inadmissible. The law deals, particularly in its criminal jurisdiction, with facts, not probabilities, and the evidence must be confined to showing that in committing the crime the one striking the fatal blow was acting under either hypnotic or post-hypnotic suggestion, and that the prisoner was the actual hypnotiser and, *ergo*, responsible.

Another point which occurs to me is the difficulty which might well arise from the trouble in getting the person hypnotised to testify in the presence of the prisoner. Might he not, by the exercise of his power over the witness, prevent him from testifying or render his testimony worthless? Yet it would not be possible to remove the prisoner and then examine the witness, for, by the inexorable rule of common law, every man is entitled to be confronted by the witness against him, and no trial can go on in the absence of the prisoner, so that if the prisoner escapes during the trial of the cause the trial must stop, the common law knowing nothing of any such proceeding as a trial *in contumatio*.

It is evident that some radical changes must be made in our criminal procedure in cases where hypnotism is alleged. I think a point has been reached where scientific investigation, traveling on well-defined and incised lines, has brought out facts that now allow some regulation of hypnotism by a change of laws. The future of the subject should now be confined to those whose training and predilections best fit them to continue research and properly instruct physician and student. Such prerequisites are absolutely necessary to place the phenomena on a dignified platform with nerve physiology. Medical schools should be able to furnish instruction to their students in this rapidly developing branch of medicine, for, as Krafft-Ebing says, "hypnotism, as a biological phenomenon of nature, offers symptoms empirically true, clear and objective, the proof of which is decisive."

We deem this matter of sufficient interest to devote the foregoing space to Dr. Howard and his critic. But we are still strongly of the opinion—a conviction that is constantly increasing in strength—that no person with perfectly healthy brain is susceptible of being hypnotised.

